# Effects of L-theanine or caffeine intake on changes in blood pressure under physical and psychological stresses

**DOI:** 10.1186/1880-6805-31-28

**Published:** 2012-10-29

**Authors:** Ai Yoto, Mao Motoki, Sato Murao, Hidehiko Yokogoshi

**Affiliations:** 1 Laboratory of Nutritional Biochemistry, School of Food and Nutritional Sciences, University of Shizuoka, 52-1 Yada, Suruga-ku, Shizuoka 422-8526, Japan

**Keywords:** L-theanine, Caffeine, Blood pressure, Acute stress, Profile of mood states

## Abstract

**Background:**

L-theanine, an amino acid contained in green tea leaves, is known to block the binding of L-glutamic acid to glutamate receptors in the brain, and has been considered to cause anti-stress effects by inhibiting cortical neuron excitation. Both L-theanine and caffeine, which green tea contains, have been highlighted for their beneficial effects on cognition and mood.

**Methods:**

In this study, we investigated the effects of orally administered L-theanine or caffeine on mental task performance and physiological activities under conditions of physical or psychological stress in humans. Fourteen participants each underwent three separate trials, in which they orally took either L-theanine + placebo, caffeine + placebo, or placebo only.

**Results:**

The results after the mental tasks showed that L-theanine significantly inhibited the blood-pressure increases in a high-response group, which consisted of participants whose blood pressure increased more than average by a performance of a mental task after placebo intake. Caffeine tended to have a similar but smaller inhibition of the blood-pressure increases caused by the mental tasks. The result of the Profile of Mood States after the mental tasks also showed that L-theanine reduced the Tension-Anxiety scores as compared with placebo intake.

**Conclusions:**

The findings above denote that L-theanine not only reduces anxiety but also attenuates the blood-pressure increase in high-stress-response adults.

## Background

To live a healthier life in so-called high-stress modern society, a growing interest in natural, minimally processed, nutritional, and healthy foods is spreading around the world, and many kinds of functional food ingredients have recently become widely used due to their health benefits. L-theanine became one of those popular items since its multiple roles in the central and autonomic nervous systems received attention. Animal studies have revealed that L-theanine affected dopamine and serotonin concentrations in the brain, underlying its anxiolytic effect [1,2]. Several reports have found increased alpha brain wave activity in humans after L-theanine administration, indicating that L-theanine could lead to a relaxed and alert state [3,4]. Kimura (2007) reported that L-theanine intake reduced heart rate and salivary immunoglobulin A responses to an acute stress task (an arithmetic task), suggesting that L-theanine could reduce stress by inhibiting cortical neuron excitation [5]. Moreover, animal studies have found that L-theanine reduced blood pressure in hypertensive rats [6,7]. It is known that stress can elevate blood pressure by stimulating the nervous system to produce large amounts of vasoconstricting hormones that increase blood pressure [8,9], L-theanine may have inhibited the increase in blood pressure through its anti-stress effects on the autonomic nervous system. From these findings, it can be hypothesized that L-theanine attenuates the stress responses in the autonomic nervous system induced by both physically and psychologically stressful tasks.

Caffeine, another major component of green tea, also has behavioral effects on autonomic nervous activities, and these effects are thought to be the opposite those of L-theanine. Caffeine is a CNS-stimulating drug that acts as an adenosine receptor antagonist in the brain [10,11]. Adenosine antagonism has been implicated as a contributor to the direct cardio-acceleratory effect of caffeine, which also increased blood pressure and respiration rate [12]. On the other hand, both caffeine and L-theanine were recently found to have beneficial effects on cognition and mood [13-15], but no study has compared these two components under conditions in which acute psychological and physical stresses increase blood pressure.

In this study, we investigated the effects of L-theanine or caffeine on mental task performance and the change in blood pressure caused by mental tasks as psychological stress and by the cold pressor test as physical stress.

## Methods

The experiment conducted in this study was approved by the research ethics committee of the University of Shizuoka, and was carried out in accordance with the Declaration of Helsinki.

### Participants

Sixteen healthy volunteers (students, eight men, eight women; ages, 22.8±2.1 years) participated in the experiment individually at similar times of the day at an interval of 7 days. The data from two women were excluded from the analyses because they were absent on at least 2 experiment days owing to temporary illness. All participants were requested to avoid eating or drinking, except for water, from 3 h before the start of each trial.

### Treatment

A cross-over, randomized, placebo-controlled design was used in this study. In total, three separate trials were performed, in which the participants orally took either L-theanine (200 mg, Taiyo Kagaku Co., Tokyo, Japan) + placebo, caffeine (100 mg, Shiratori Pharmaceutical Co., Chiba, Japan) + placebo, or placebo only on each day. Dextrin (Nisshin Pharma Inc., Tokyo, Japan) was used as the placebo. All sample capsules were taken with 250 mL warm water at about 25°C. Treatments were allocated using a Latin square design such that the order of treatments was counterbalanced across participants.

Yokogoshi et al. [(1998)] reported that L-theanine increased by 1 h at the latest in the serum, the liver, and the brain after administration, and thereafter decreased sharply in the serum and liver [1,16]. Van der Pijl et al. [(2010)] reported that L-theanine plasma concentration reached the peak between 32 and 50 min after oral ingestion, and its half-life ranged from 58 min to 74 min in humans [17]. Terashima et al. [(1999)]. also reported that L-theanine could influence the secretion and function of neurotransmitters in the central nervous system even at 30 min after oral administration [18]. On the other hand, caffeine absorption from the gastrointestinal tract is rapid and reaches 99% in about 45 min after ingestion, while peak plasma caffeine concentration is reached between 15 min and 120 min, and half-life ranges from 2.5 h to 4.5 h after oral ingestion in humans [19]. To allow a peak of both L-theanine and caffeine appears during the stress load period, sample treatment was decided to be taken at 36 min before the end of the mental tasks session (DT and AT as defined below), followed by subjective assessment which was performed from 38 min to 43 min, physiological measurement from 44 min to 45 min, and physical stress task session (CPT) from 45 min to 49 min after the sample treatment.

### Stress load task

After each sample was taken, an auditory oddball target detection task (DT) lasting for 5 min each and an arithmetic mental task (AT) lasting for 10 min each were both imposed twice as the psychological stress load. In the DT, participants were required to click the left button of a computer mouse as quickly as possible to target stimuli (a single tone of 2,000 Hz lasting for 0.1 s) that occur infrequently and irregularly within a series of standard stimuli (a single tone of 1,000 Hz lasting for 0.1 s). The AT required participants to add two numbers (each from 1 to 9) that were being displayed on a PC monitor and to enter the answer through the keyboard quickly and accurately. The number and accuracy of the answers to the second AT, which was taken from 26 min to 36 min after each sample intake, were used for data analysis.

A cold pressor test (CPT) was taken to establish physical acute stress [20]. Participants were asked to immerse their right hand, past the level of the wrist, for 1 min in a bucket filled with slushy ice water (1.5±0.3C) and then to place the hand on the table nearby with a towel underneath the hand.

### Subjective assessment

The Profile of Mood States (POMS) and the visual analogue scales (VAS) for subjective ratings on mood state were also completed before the intake as a basic control and after all of the mental tasks were finished.

The short version of POMS was used to assess distinct affective mood states. POMS is a popular tool that is widely used among psychologists and scientists in many fields. Six identifiable mood or affective states can be measured and were used for analysis in this study: Tension-Anxiety (T-A), Depression-Dejection (D), Anger-Hostility (A-H), Vigor-Activity (V), Fatigue-Inertia (F), and Confusion-Bewilderment (C).

VAS comprised five scales including feelings of fatigue, relaxation, arousal, pressure, and tension. At the end of each trial, the subjects used the scales to rate their painful feelings about accomplishing the CPT and their feelings of annoyance about DT and AT.

### Physiological measurement

Arterial pressure in each participant’s left thumb was recorded continuously by Finometer Pro (FMS, Finapres Measurement Systems, Arnhem, the Netherlands). Simultaneously, skin temperature of the back of the left hand was recorded using a BioAmplifier (Polymate AP1132, TEAC, Tokyo, Japan). The sampling rate was 200 Hz. As baseline data, both the blood pressure and skin temperature were measured for 1 min before the intake. Measurement after mental tasks (AMT) was also made for 1 min at 44 min after the intake of each sample, followed by measurement for 4 min after CPT was started.

Baseline data were calculated by averaging the 1 min data before each intake. Differences in blood pressure and skin temperature from the baseline were calculated using the mean value of every 10-s epoch for the above measurements after intake. The first 10-s epoch of the AMT was described as AMT1, and the second, third, fourth, fifth, and sixth 10-s epochs were described as AMT2, AMT3, AMT4, AMT5, and AMT6, respectively. Similarly, CPT1 to CPT6 for the CPT epochs, and RP1 to RP18 for epochs during the 3-min recovering period after the 1 min CPT were named respectively and used for the analysis.

### Procedure

**Figure 1:**
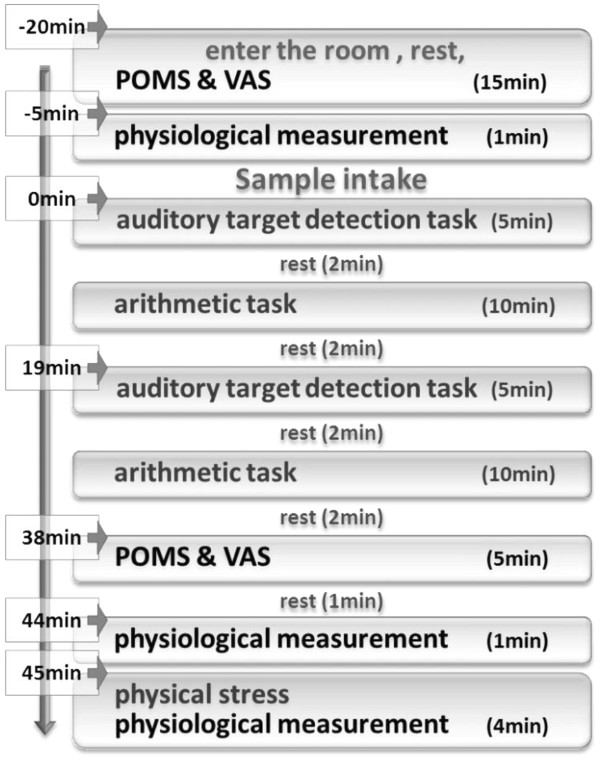
The experimental procedure.

Figure 1 shows the experimental procedure. Each participant was required to attend a total of 3 study days, which were conducted 7 days apart, to ensure a sufficient washout between conditions. Prior to the start of the experiment, all participants were given the opportunity to familiarize themselves with all of the stress load tasks. The experiments took place in a quiet room. The room temperature was 26.4±1.1°C, and the humidity was 51.5±6.8%. On each experiment day, each participant entered the room and rested for 15 min. During the resting time, a skin-surface temperature probe was attached, and POMS and VAS were completed. After the rest, a 1-min physiological measurement session to obtain baseline data took place, followed by sample treatment. After the oral administration, mental tasks were performed: DT (5 min), rest (2 min), AT (10 min), and rest (2 min); the cycle was then repeated. Then, POMS and VAS and another 1-min measurement were completed again to obtain data after the mental tasks. CPT for 1 min was then started. At the same time, measurement was recorded for 4 min (1 min for CPT, 3 min for RP after CPT). At last, VAS about feelings of DT, AT, and CPT was completed.

### Statistical analysis

Data were analyzed using IBM SPSS Statistics version 19. Prior to the primary statistical analysis, separate, one-way, repeated measures ANOVAs of the baseline data were conducted to ascertain any chance baseline differences across study days prior to the treatments.

L-theanine reduced blood pressure in spontaneously hypertensive rats but not in rats with normal blood pressure [6,7]. Thus it is considerable that L-theanine might act in different ways between people in whom stress increases whose blood pressure in different ways. With this in mind, we divided the participants into two groups after the experiment according to their changes in systolic blood pressure after the mental tasks in the placebo intake condition. The half of participants who showed greater than average changes in blood pressure were sorted into a high-response group and the other half into a low-response group.

Differences in blood pressure and skin temperature from the basic control were calculated and used for a repeated-measures ANOVA with group (high-response group and low- response group), treatment (L-theanine, caffeine, and placebo), and epoch (six epochs for AMT, CPT and 18 epochs for CPT). Repeated-measures ANOVA with group and treatment was also applied to the task performance data. A Tukey’s honestly significant difference (HSD) *post hoc* test was applied to data groups with significant main effect (*P* <0.05). Differences in POMS and VAS scores were analyzed using the nonparametric Friedman test to detect differences in treatments. The Wilcoxon signed rank test was further carried out to evaluate the changes among treatments.

## Results

### Systolic blood pressure

**Figure 2:**
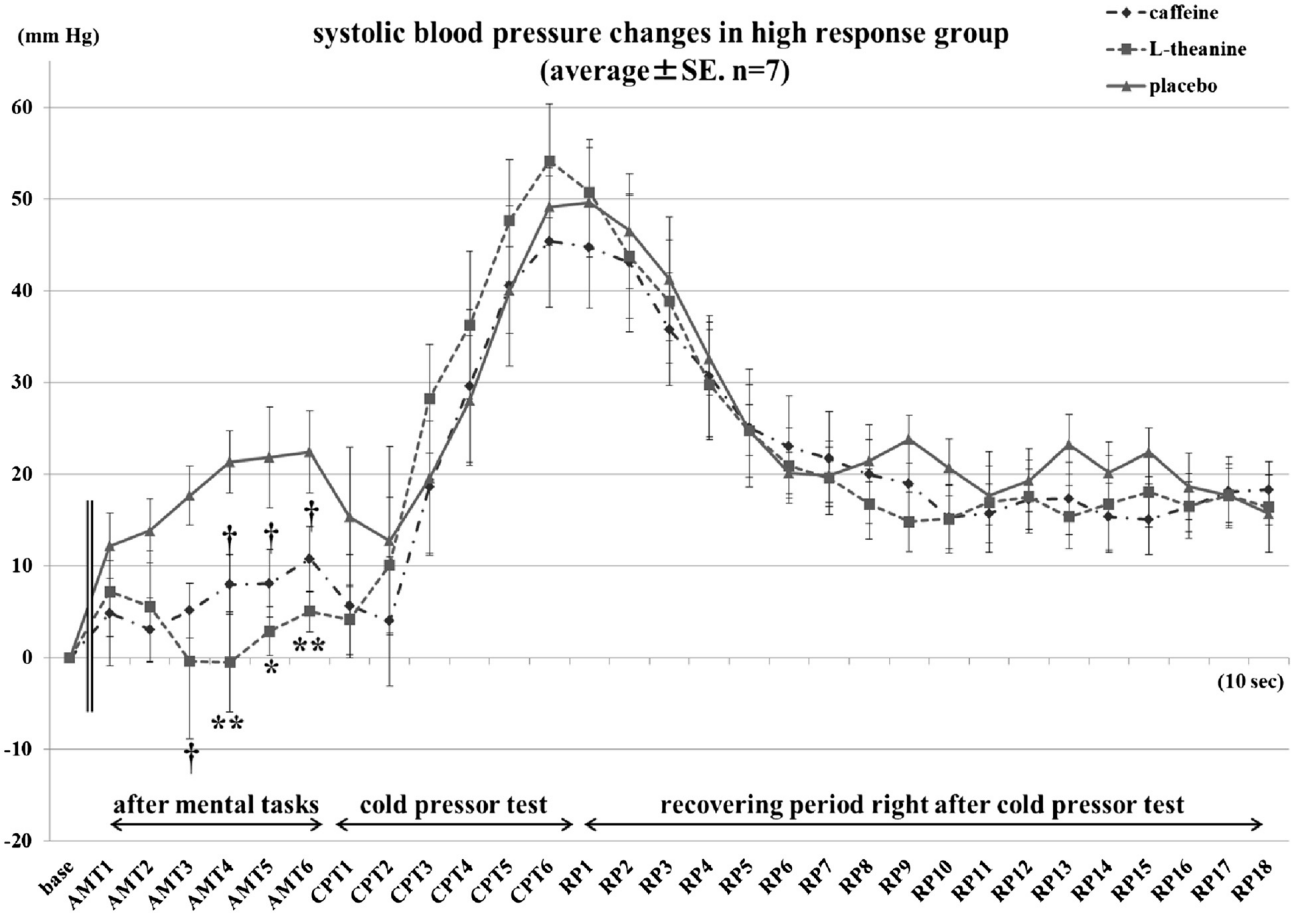
**Systolic blood pressure caused by mental tasks and cold pressor test in the high-response group.** The base was calculated by averaging the 1-min data before intakes. AMT1 represents the first 10-s epochs of the measurements taken after mental tasks. Similarly, the second through sixth 10-s epochs of the measurement are called AMT2, AMT3, AMT4, AMT5, and AMT6, respectively, CPT1 to CPT6 for epochs of measurement during the cold pressor test, and RP1 to RP18 for epochs during the 3-min recovery period after CPT (†=*P* <0.1, *=*P* <0.05, **=*P* <0.01).

Changes in systolic blood pressure and diastolic blood pressure are summarized in Table 1. In the AMT period, there was an interaction effect between treatment and group (F(2,24)=3.438, *P*=0.049). The high-response group revealed main effects of treatment significantly at AMT4, AMT5, and AMT6 and showed a trend at AMT3 (F(2,12)=6.958, 5.500, 7.195, and 2.994, *P*=0.010, 0.020, 0.009, 0.088). As shown in Figure 2, the results of Tukey’s LSD showed that in the 1-min measurement of the high-response group after the mental tasks, the L-theanine intake condition tended to decrease the systolic blood pressure in the AMT3 period (*P*=0.082), and showed a significant effect of lower value in the AMT4, AMT5, and AMT6 periods compared with that of the placebo intake condition (*P*=0.008, 0.019, 0.008). Caffeine intake showed a trend of lower systolic blood pressure than the placebo condition only at AMT4, AMT5, and AMT6 (*P*=0.099, 0.090, 0.068).

**Table 1:** Changes in systolic blood pressure and diastolic blood pressure

**Systolic blood pressure changes (mmHg)**												
	**High-response group**						**Low-response group**					
	**Mean blood pressure**			**SEM**			**Mean blood pressure**			**SEM**		
	**Caffeine**	**L-theanine**	**Placebo**	**Caffeine**	**L-theanine**	**Placebo**	**Caffeine**	**L-theanine**	**Placebo**	**Caffeine**	**L-theanine**	**Placebo**
Base	0	0	0	0	0	0	0	0	0	0	0	0
AMT1	4.83	7.17	12.17	5.75	4.85	3.58	10.32	5.78	6.82	3.02	7.25	3.12
AMT2	3.05	5.55	13.82	3.48	6.04	3.48	5.62	2.07	1.36	3.84	5.23	2.06
AMT3	5.10	−0.36	17.68	2.95	8.50	3.24	5.78	4.31	3.98	2.89	5.80	2.01
AMT4	7.95	−0.49	21.35	3.24	5.45	3.36	5.98	9.05	3.64	5.19	4.79	2.39
AMT5	8.08	2.88	21.82	3.71	2.66	5.46	6.92	9.28	2.75	4.25	5.35	2.56
AMT6	10.75	5.01	22.41	3.55	2.21	4.47	5.91	9.98	3.28	4.47	5.75	2.63
CPT1	5.63	4.14	15.34	5.61	3.78	7.61	−2.63	7.00	3.19	5.17	7.55	4.03
CPT2	3.98	10.10	12.74	7.04	7.37	10.28	−9.26	−2.76	3.42	5.66	6.86	5.74
CPT3	18.58	28.22	19.57	7.20	5.92	8.47	3.98	12.31	15.95	5.00	7.35	4.41
CPT4	29.62	36.24	28.04	8.36	8.02	7.11	16.20	23.71	25.82	4.58	5.79	3.57
CPT5	40.52	47.64	40.05	8.71	6.67	4.70	31.65	32.08	33.61	5.23	7.46	4.99
CPT6	45.36	54.15	49.15	7.15	6.22	4.24	38.10	39.48	39.24	6.85	7.55	5.25
RP1	44.70	50.71	49.64	6.61	5.76	5.98	35.92	40.74	35.55	4.57	7.75	6.34
RP2	43.03	43.70	46.51	7.48	6.69	6.26	35.18	43.70	36.82	5.86	7.85	6.26
RP3	35.78	38.82	41.28	6.13	6.70	6.77	31.30	33.81	33.41	4.95	6.94	6.57
RP4	30.66	29.77	32.61	6.63	6.02	4.00	26.18	26.68	26.41	4.76	6.68	4.66
RP5	25.05	24.68	24.78	6.41	5.04	2.77	24.11	20.12	21.92	4.78	5.82	4.89
RP6	22.99	20.92	20.12	5.56	4.09	2.23	20.73	18.22	17.99	4.70	5.20	4.58
RP7	21.68	19.58	19.90	5.15	3.96	3.03	16.51	21.51	18.14	3.77	5.38	4.31
RP8	19.99	16.77	21.42	5.33	3.83	2.28	15.97	19.44	17.05	4.62	5.35	2.91
RP9	18.95	14.82	23.78	4.52	3.25	2.61	15.17	17.75	18.78	4.82	5.95	3.62
RP10	15.32	15.12	20.72	3.46	3.71	3.11	13.72	18.22	20.75	5.11	6.80	3.90
RP11	15.72	16.94	17.67	3.20	5.49	3.23	15.27	18.48	22.31	4.22	6.66	4.34
RP12	17.25	17.55	19.34	3.29	3.98	3.38	17.28	18.84	21.79	4.70	6.35	4.66
RP13	17.35	15.34	23.24	3.92	3.46	3.27	15.42	22.60	17.48	4.96	6.25	4.65
RP14	15.36	16.77	20.18	3.67	5.27	3.30	14.40	18.04	15.75	5.45	5.50	3.33
RP15	15.06	18.07	22.38	3.85	3.84	2.66	9.80	20.72	15.35	5.23	6.03	3.03
RP16	16.48	16.54	18.65	2.71	3.50	3.63	12.42	23.12	17.28	4.57	7.01	3.83
RP17	18.10	17.70	17.67	3.75	2.96	3.49	13.92	19.35	18.24	4.87	5.63	3.76
RP18	18.33	16.40	15.70	3.04	1.96	4.23	14.43	16.14	19.36	3.91	6.94	3.10
**Diastolic blood pressure changes (mmHg)**												
	**High-response group**						**Low-response group**					
	**Mean blood pressure**			**SEM**			**Mean blood pressure**			**SEM**		
	**Caffeine**	**L-theanine**	**Placebo**	**Caffeine**	**L-theanine**	**Placebo**	**Caffeine**	**L-theanine**	**Placebo**	**Caffeine**	**L-theanine**	**Placebo**
Base	0	0	0	0	0	0	0	0	0	0	0	0
AMT1	4.96	6.00	11.74	3.66	2.30	1.97	7.41	5.87	7.14	2.65	3.20	1.62
AMT2	4.47	5.34	12.30	2.53	1.31	3.56	2.38	1.46	4.14	2.61	2.98	2.08
AMT3	5.09	0.61	12.60	1.98	3.27	2.91	3.51	3.00	4.39	2.71	3.27	2.27
AMT4	5.73	2.14	17.07	2.39	2.20	2.83	4.22	5.31	5.39	3.34	3.22	2.00
AMT5	5.81	5.67	16.10	2.27	3.43	4.83	5.39	4.99	4.51	2.92	3.03	1.88
AMT6	7.19	6.64	16.90	2.37	3.08	3.73	3.95	6.66	4.64	2.98	3.03	1.73
CPT1	7.70	7.64	18.30	3.54	3.66	4.12	3.16	10.21	7.52	4.15	3.32	2.35
CPT2	8.56	9.58	16.31	3.87	3.60	5.78	0.89	1.36	6.49	4.13	3.14	2.88
CPT3	20.21	22.98	22.82	4.57	4.24	3.62	9.71	11.34	16.82	3.16	3.73	1.73
CPT4	28.40	30.20	29.32	5.15	6.15	1.49	18.34	20.10	23.91	2.86	2.84	2.14
CPT5	36.06	38.60	38.72	5.17	5.60	2.20	24.56	26.83	28.84	2.47	3.38	1.91
CPT6	39.39	42.83	43.18	4.30	4.62	3.75	27.31	30.29	32.04	2.90	3.73	2.23
RP1	35.79	38.60	42.20	5.27	4.67	4.80	24.09	27.87	27.85	2.71	3.48	2.61
RP2	32.09	31.23	36.65	6.64	5.16	4.47	19.93	25.49	24.41	3.35	3.74	2.22
RP3	25.97	26.03	31.75	5.68	5.50	4.00	16.69	18.37	19.62	3.04	3.84	3.18
RP4	21.84	21.50	25.34	5.36	5.65	2.92	13.60	15.34	16.02	3.16	3.82	2.84
RP5	18.16	17.67	19.81	5.31	4.85	2.57	12.40	12.10	12.68	3.04	3.10	2.99
RP6	16.11	15.78	16.97	4.49	4.38	1.68	10.79	11.10	11.09	3.20	2.71	2.72
RP7	14.83	14.55	15.90	3.73	4.37	1.58	8.04	12.81	11.44	2.59	2.66	2.59
RP8	13.71	12.40	16.55	3.80	3.67	1.98	8.26	10.90	10.98	3.29	2.75	1.79
RP9	12.59	12.25	18.04	3.83	3.38	2.17	6.76	10.14	12.08	3.11	2.99	2.36
RP10	9.06	12.00	15.18	2.98	3.86	2.63	5.95	10.23	12.16	3.47	2.99	2.64
RP11	10.23	12.78	13.37	2.58	3.72	2.73	7.45	10.24	12.82	2.42	2.62	2.38
RP12	10.90	12.94	14.80	2.89	3.04	2.93	7.04	10.61	12.76	3.19	3.18	2.62
RP13	10.04	11.61	16.50	3.04	2.74	2.47	6.49	12.07	9.99	2.90	2.95	2.94
RP14	8.90	12.14	13.94	2.90	3.26	2.53	5.72	8.90	9.75	3.19	3.04	1.67
RP15	9.30	12.54	16.81	3.12	2.76	1.97	4.38	11.49	8.88	3.33	2.78	1.70
RP16	8.99	10.44	13.04	2.50	1.71	2.86	5.96	11.91	9.58	2.28	2.87	1.76
RP17	9.30	11.80	12.77	2.69	2.30	3.38	5.79	9.37	10.08	3.29	2.70	2.12
RP18	9.66	12.08	10.03	2.33	2.53	3.43	6.17	8.93	11.50	2.43	3.06	2.57

In the rest periods, systolic blood pressure did not differ significantly among treatments.

**Figure 3:**
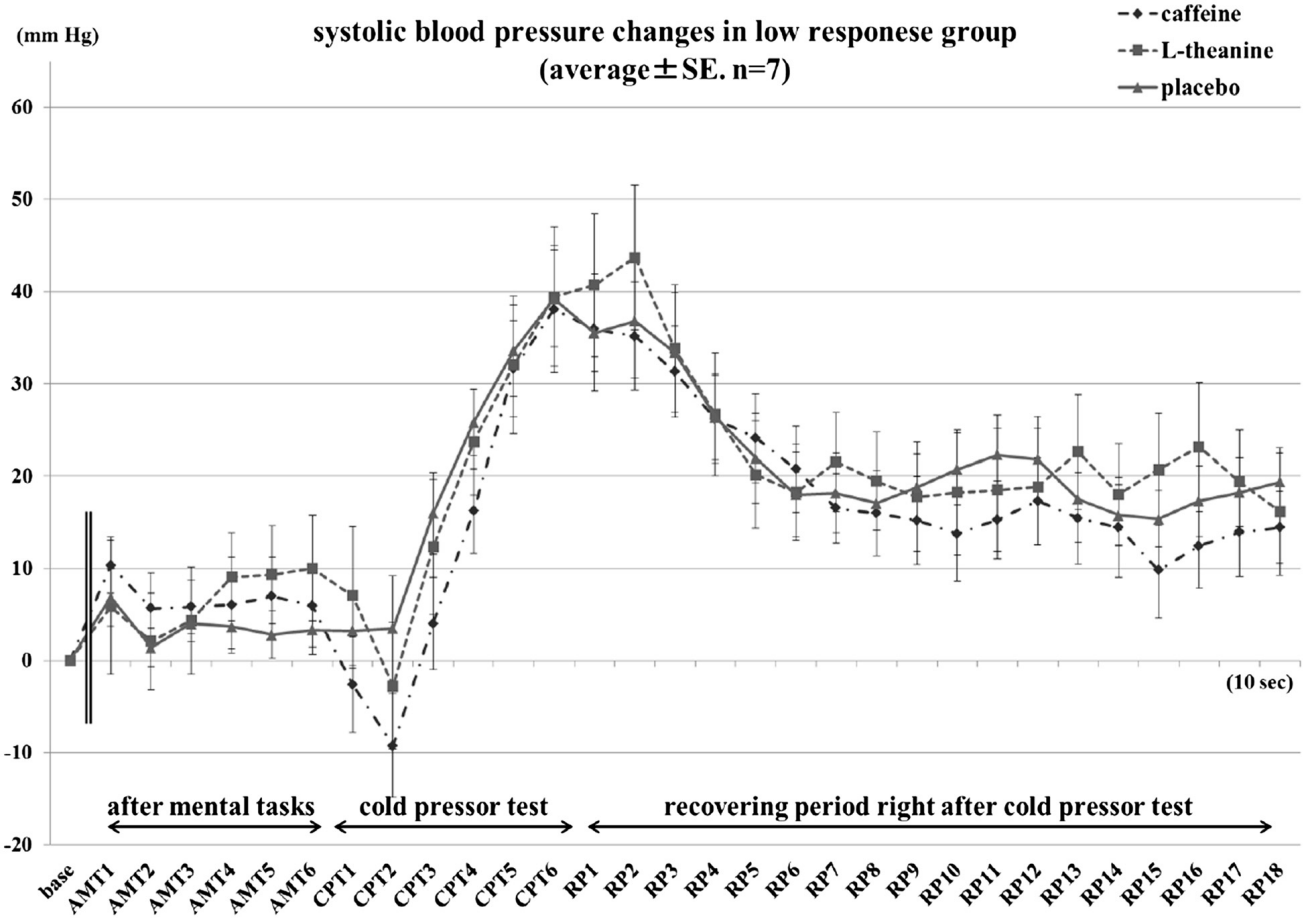
**Systolic blood pressure caused by mental tasks and cold pressor test in the low-response group.** The base was calculated by averaging the 1-min data before intakes. AMT1 represents the first 10-s epochs of the measurements taken after the mental tasks. Similarly, the second through sixth 10-s epochs of the measurement are called AMT2, AMT3, AMT4, AMT5, and AMT6, respectively, CPT1 to CPT6 for epochs of measurement during the cold pressor test, and RP1 to RP18 for epochs during the 3-min recovery period after CPT.

No treatment effect was found in the low-response group (Figure 3).

### Diastolic blood pressure

Diastolic blood pressure in the AMT period revealed trends for the main effect of treatment (F(2,24)=2.577, *P*=0.097) and of group (F(1,12)=3.361, *P*=0.092). In the high-response group, treatment affected diastolic blood pressure at AMT4 and AMT6 (F(2, 12)=7.932, 4.300, *P*=0.006, 0.039), and lower values were obtained by L-theanine (*P*=0.006, 0.056) or caffeine intake (*P*=0.033, 0.071) than in the placebo condition. Diastolic blood pressure did not differ significantly among the treatments in the other periods

No treatment effect was found in the low-response group in any of the periods of the blood pressure measurements.

### Skin temperature

Skin temperature was not affected by the different sample intakes in each group or two groups together in this study (data not shown).

### POMS and VAS

**Figure 4:**
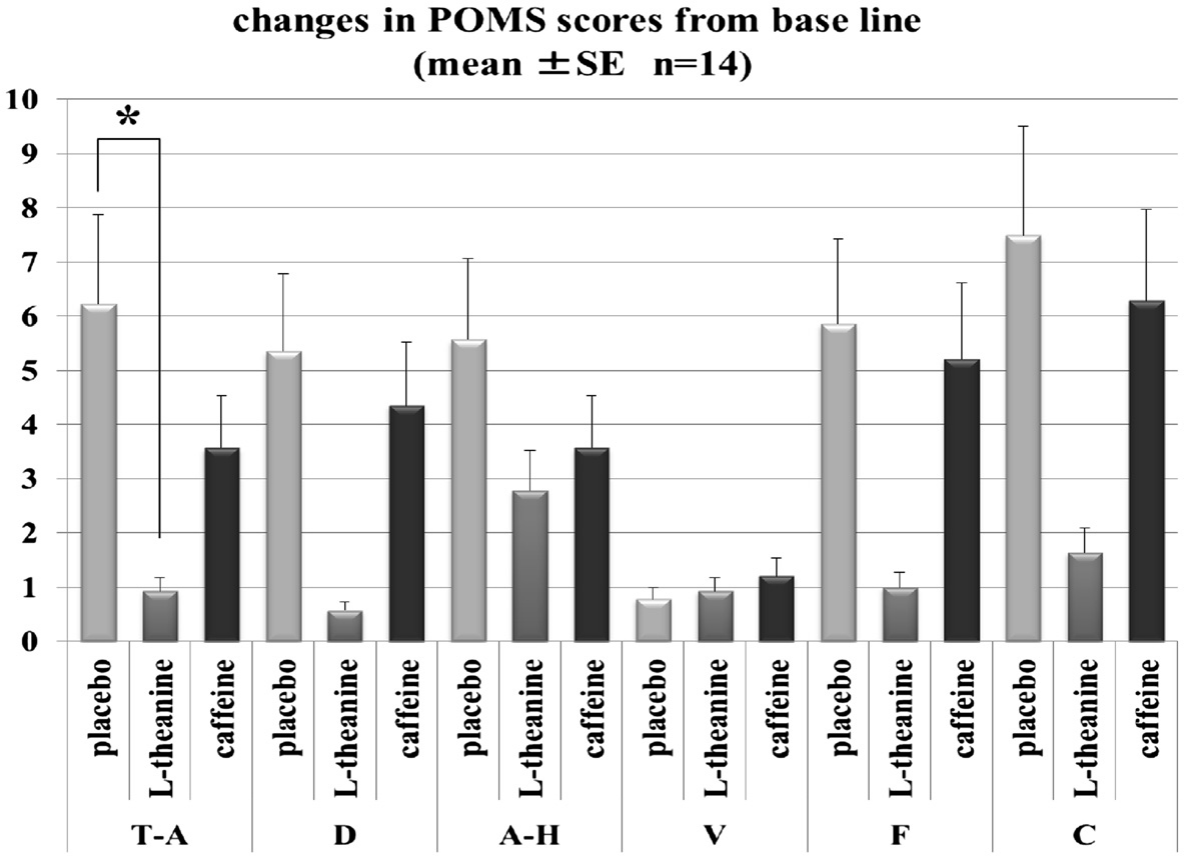
**Changes in POMS scores from the baseline after mental tasks.** A-H, Anger-Hostility; C, Confusion-Bewilderment; D, Depression-Dejection; F, Fatigue-Inertia; T-A, Tension-Anxiety; V, Vigor-Activity.

Figure 4 presents the significant results of POMS scores. T-A scores and A-H scores showed treatment effects over the two groups together (χ^2^=6.000, 6.048, *P*=0.050, 0.049), and L-theanine intake decreased T-A score below that in the placebo condition (*P*=0.004).

No difference was obtained among treatments in each group or two groups together for VAS assessments.

### Task performance

There was no interaction effect between group and treatment. Over two groups together, treatment tended to affect the number of answers in AT (F(2,26)=3.261, *P*=0.054), and participants answered more questions after caffeine intake than after placebo intake (*P*=0.052). There was no effect on the accuracy of the answers.

## Discussion

Oral administration of L-theanine significantly changed both systolic and diastolic blood pressures in the high-response group during the latter part of AMT compared with the placebo condition. These results demonstrated the possibility that L-theanine can attenuate blood pressure elevation induced by mental tasks. This finding agreed with the blood pressure-reducing effect of L-theanine intake reported in another study, in which theanine inhibited the blood-pressure increase resulting from caffeine intake [15]. The AMT measurements were carried out after participants finished all of the mental tasks and just before the CPT. That is to say, the participants felt stressed not only by the mental tasks but also by, or even more by, their knowledge that their CPT would be taken in a minute. The high-response group included participants who showed large increases in mean systolic blood pressure after the mental tasks in the placebo intake condition, and the range of elevation was 9.46 to 33.88 mmHg. It has been considered that young adults who show a large blood-pressure response to psychological stress may be at risk for hypertension as they approach mid-life [9]. From this point of view, participants in the high-response group in this study might be at risk of hypertension. The result showed that the intake of 200 mg L-theanine significantly attenuated the blood pressure response caused by psychological stress in the high-response group. This indicated that L-theanine reduced blood pressure not only for spontaneously hypertensive rats [6,7] but also for humans at risk of hypertension, despite the lower dose of L-theanine (200/62.8=3.2 mg/kg body weight) for the high-response group comparing with 2,000 mg/kg for hypertensive rats. The mechanism underlying this result might be the same as that reported in Kimura et al. [(2007)], that L-theanine could cause anti-stress effects by inhibiting cortical neuron excitation, which attenuates the sympathetic nervous activation response to the acute stress task [5].

Stress may not directly cause hypertension, but it can lead to repeated blood pressure elevations, which can eventually lead to hypertension [8]. With this in mind, L-theanine might be useful for preventing the development of hypertension. Although we could not obtain results of this anti-stress effect from the VAS assessment, the results of POMS scores in T-A indicated that L-theanine intake improved participants’ mood by lowering the tension and anxiety caused by psychological stress. This supported the relaxing effect reported in Juneja et al. [(1999)] that L-theanine can promote the generation of alpha brain waves and induce a relaxed state in humans approximately 40 min after intake [3].

Contrary to our hypothesis, caffeine also tended to inhibit blood-pressure elevation in this study, and it did not show opposite effects to L-theanine on blood pressure raised by psychological stress. Suleman and Siddiqui (1997 to 2004) suggested that caffeine raised blood pressure during stress by elevating the resting baseline from which the response was measured and not by potentiating the acute blood pressure stress response [12]. The psychological stress load used in the current study started right after the sample intake without resting period, which might have been strong and thus potentiated the stress response to a level higher than the response potentiated by caffeine intake. Moreover, Lane and Williams (1987) reported that caffeine potentiated stress-related increases in forearm vasodilation [21]. This might also lower the raised blood pressure measured from the thumb of the left hand in our study.

On the other hand, neither L-theanine nor caffeine decreased the rise in blood pressure caused by CPT compared with the placebo. This might be attributable to the difference in the mechanism between blood pressure elevation by psychological stress and that by the physical stress of pain. Further studies are needed to confirm this and to investigate how L-theanine or caffeine influences the autonomic nervous system responses under other kinds of physical stress.

At last, due to the limitation on the amount of female participants in this study, the possible effects of their menstrual period are difficult to be discussed this time. Thus, there is a possibility that the results might be different if the number of participants is large enough to sort them into four groups: two male groups (the high- and the low-response groups) and two female groups (also the high- and low-response groups). We would like to confirm this with larger numbers of both male and female participants in future.

## Conclusions

Our results suggested that L-theanine not only reduces anxiety but also attenuates the rise in blood pressure in high-stress-response adults. In addition, neither L-theanine nor caffeine showed any effect on decreasing the rise in blood pressure caused by strong physical stress, such as the CPT used in this study.

## Abbreviations

A-H: Anger-hostility; AMT: Measurement after mental tasks; ANOVA: Analysis of variance; AT: Arithmetic mental task; C: Confusion-bewilderment; CPT: Cold pressor test; D: Depression-dejection; DT: Auditory oddball target detection task; F: Fatigue-inertia; HSD: Tukey’s honestly significant difference; POMS: Profile of Mood States; T-A: Tension-anxiety; V: Vigor-activity; VAS: Visual analogue scales.

## Competing interests

The authors declare that they have no competing interests.

## Authors’ contributions

AY conceived and designed the study, performed the experiments and the statistical analysis, and drafted the manuscript. MM and SM helped to carry out the experiments and to perform data analysis. HY conceived of the study, participated in its design and coordination, and helped to draft the manuscript. All authors have read and approved the final manuscript.
